# Pyrene-Benzimidazole Derivatives as Novel Blue Emitters for OLEDs

**DOI:** 10.3390/molecules26216523

**Published:** 2021-10-28

**Authors:** Thenahandi Prasanthi Deepthika De Silva, Sang Gil Youm, Frank R. Fronczek, Girija Sahasrabudhe, Evgueni E. Nesterov, Isiah M. Warner

**Affiliations:** 1 Department of Chemistry, Louisiana State University, Baton Rouge, LA 70803, USA; tpd005@shsu.edu (T.P.D.D.S.); ffroncz@lsu.edu (F.R.F.); Girija.Sahasrabudhe@newcastle.edu.au (G.S.); 2 Department of Chemistry and Biochemistry, Northern Illinois University, DeKalb, IL 60115, USA; syoum@umn.edu (S.G.Y.); een@niu.edu (E.E.N.)

**Keywords:** OLED, benzimidazole, pyrene, electroluminescence, optoelectronic applications

## Abstract

Three novel small organic heterocyclic compounds: 2-(1,2-diphenyl)-1*H*-benzimidazole-7-*tert*-butylpyrene (compound **A**), 1,3-di(1,2-diphenyl)-1*H*-benzimidazole-7-*tert*-butylpyrene (compound **B**), and 1,3,6,8-tetra(1,2-diphenyl)-1*H*-benzimidazolepyrene (compound **C**) were synthesized and characterized for possible applications as blue OLED emitters. The specific molecular design targeted decreasing intermolecular aggregation and disrupting crystallinity in the solid-state, in order to reduce dye aggregation, and thus obtain efficient pure blue photo- and electroluminescence. Accordingly, the new compounds displayed reasonably high spectral purity in both solution- and solid-states with average CIE coordinates of (0.160 ± 0.005, 0.029 ± 0.009) in solution and (0.152 ± 0.007, 0.126 ± 0.005) in solid-state. These compounds showed a systematic decrease in degree of crystallinity and intermolecular aggregation due to increasing steric hindrance, as revealed using powder X-ray diffraction analysis and spectroscopic studies. An organic light-emitting diode (OLED) prototype fabricated using compound **B** as the non-doped emissive layer displayed an external quantum efficiency (EQE) of 0.35 (±0.04)% and luminance 100 (±6) cd m^−2^ at 5.5 V with an essentially pure blue electroluminescence corresponding to CIE coordinates of (0.1482, 0.1300). The highest EQE observed from this OLED prototype was 4.3 (±0.3)% at 3.5 V, and the highest luminance of 290 (±10) cd m^−2^ at 7.5 V. These values were found comparable to characteristics of the best pure blue OLED devices based on simple fluorescent small-molecule organic chromophores.

## 1. Introduction

Organic light emitting diodes (OLEDs) have been steadily present over the past 30 years, from a laboratory concept stemming from the pioneering work of Ching W. Tang and Steven A. Van Slyke, to a leading technology in the consumer electronics market [1,2]. When compared to competing liquid crystal display (LCD) technology, OLED displays offer advantages such as energy conservation, device mechanical flexibility needed for curved electronic displays with broad viewing angles, outstanding picture quality due to the absence of back-lighting, and small overall device thickness with significantly lower weight [2,3]. The much shorter lifespan of OLED panels as compared to that of current market leader LCDs, however, is still a formidable challenge, stemming in major part from inferior performance of blue emitters [2,3,4]. Blue OLED emitters typically suffer from inefficient charge injection and mobility, lower photo/thermal/chemical stability, and insufficient spectral purity as compared to red and green emitters in full-color RGB (red, green, blue) electronic displays [4,5,6] The intrinsically wide HOMO-LUMO energy gaps of blue emitters make charge injection from the electrodes/supporting organic layers to the emissive layer more difficult. In addition, blue emitters are susceptible to rapid degradation as a result of side-reactions from the high-energy excited state [4]. Therefore, it is important to continue developing novel blue emitters that can potentially address these inadequacies [4,5,6,7].

Several strategies are suggested to address the aforementioned inadequacies of blue emitters. Such efforts include optimization of molecular design, OLED architecture, and the exciton harvesting mechanism [4,5]. Interestingly, multifunctional molecular designs are recognized as one of the most promising solutions. In this regard, hole and/or electron transport moieties may be incorporated into blue emitters in order to facilitate proper flux of charges to the emissive layer [4]. Multifunctional emitters are classified as *p*-type (hole transporters) [8,9,10,11], *n*-type (electron transporters) [12,13,14], and bipolar (electron and hole transporters) [15,16,17]. The use of multifunctional emissive materials could effectively reduce the number of supporting organic semiconducting layers in OLEDs, e.g., charge injectors and transporters, thus lowering the complexity and cost of OLED devices [4]. 

**Figure 1:**
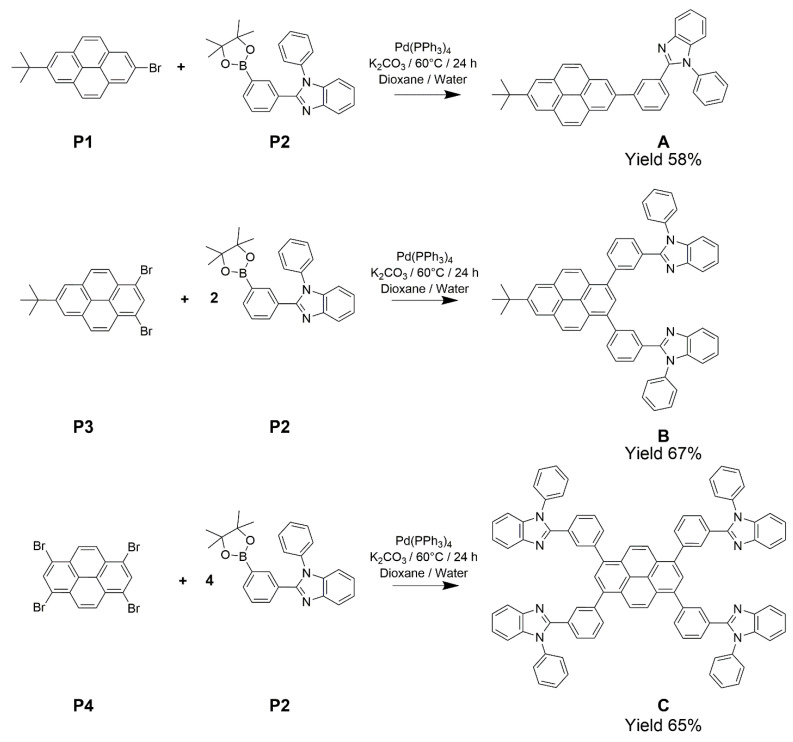
Synthesis and structure of pyrene-benzimidazole derivatives, 2-(1,2-diphenyl)-1*H*-benzimidazole-7-*tert*-butylpyrene (compound **A**), 1,3-di(1,2-diphenyl)-1*H*-benzimidazole-7-*tert*-butylpyrene (compound **B**), and 1,3,6,8-tetra(1,2-diphenyl)-1*H*-benzimidazolepyrene (compound **C**).

In this manuscript, we report the design, synthesis, and characterization of three novel, structurally similar, multifunctional small organic molecules. These compounds are hybrids of pyrene and benzimidazole derivatives, namely, 2-(1,2-diphenyl)-1*H*-benzimidazole-7-*tert*-butylpyrene (compound **A**), 1,3-di(1,2-diphenyl)-1*H*-benzimidazole-7-*tert*-butylpyrene (compound **B**), and 1,3,6,8-tetra(1,2-diphenyl)-1*H*-benzimidazolepyrene (compound **C**), presented in Figure 1. In designing these compounds, pyrene moieties were chosen to serve as blue luminophores due to a number of favorable characteristics. In particular, pyrene functional groups show resistance to photo- and thermal-degradation related to the high chemical stability of the polyaromatic hydrocarbon (PAH) core, high fluorescence quantum efficiency, favorable charge carrier properties, ease of synthesis/modification, and low cost [7,18]. Due to these favorable characteristics, a vast number of pyrene derivatives have been studied as emissive materials, charge injection materials, and charge transport materials in OLEDs [7,18]. However, pyrene-derived pure blue OLED emitters are less common owing to extensive π-π stacking of the nearly planar pyrene moieties in the condensed state, resulting in excimer formation. This phenomenon produces a substantial bathochromic shift in the pyrene derivatives’ emission spectrum, leading to greenish-blue, bluish-green, or green emission in thin films [7]. In addition, pyrene aggregation accounts for aggregation-caused quenching of the fluorescence emission, thus reducing the OLED emission efficiency [3,7]. 

There are notable exceptions to the common aggregation-caused quenching situation. One example includes pyrene derivatives that exhibit aggregation induced emission (AIE) [19,20]. Compounds displaying AIE phenomenon show enhanced emission in the solid-state as a result of restricted intra-molecular motion. Strategies other than AIE to preserve desirable optical characteristics of small-molecule pyrene derivatives include using twisted structures to restrict π-π stacking for relatively small molecules [21,22], designing polymers/oligomers/dendrimers with pyrene moieties placed in such a way that they cannot aggregate effectively due to steric effects [23,24], and applying a suitable host matrix to dilute pyrene derivative and diminish dye aggregation [25]. The novel compounds discussed in this manuscript, however, are not AIE materials. Instead, our design utilizes multiple phenyl and/or tertiary butyl groups attached to the pyrene-benzimidazole cores of compounds **A**, **B**, and **C**. These groups were found to effectively reduce π-π stacking of pyrenyl moieties in the solid-state by causing substantial steric hindrance. In addition to further inducing steric hindrance, electron deficient benzimidazole moieties in the target compounds were added in order to facilitate electron transport since these units are known to possess electron transporting properties, particularly when conjugated to organic or transition metal electron donors [16,26,27]. 

Compounds **A**, **B**, and **C** were systematically evaluated for morphological, photo-thermal, optical, and electrochemical properties to assess the suitability of these molecules as blue OLED emitters. As expected, compounds **A**, **B**, and **C** showed systematic reduction in degree of crystallinity due to increasing steric hindrance that prevents solid-state crystalline packing of these compounds. As a result, an essentially pure blue emission was observed from all three compounds in both solution and solid states. The spectroscopic characteristics of these compounds were thoroughly investigated in both solution and solid states. Since compound **B** showed the most suitable photophysical properties among the three compounds investigated, a non-doped OLED prototype was fabricated using compound **B** as the emissive material. As expected, this OLED prototype showed an essentially pure blue electroluminescence with Commission Internationale de L’Eclairage (CIE) coordinates of (0.148, 0.130). Other OLED performance characteristics including power and current efficiencies and external quantum efficiency (EQE) were also evaluated for this prototype. 

## 2. Experimental Section

### 2.1. Materials

Tetrakis(triphenylphosphine)palladium(0), 2-bromo-7-*tert*-butylpyrene, 1,3-dibromo-7-*tert*-butylpyrene, 1,3,6,8-tetrabromopyrene, 1-phenyl-2-[3-(4,4,5,5-tetramethyl-1,3,2-dioxaborolan-2-yl)phenyl]-1*H*-benzimidazole, and 2,6-dibromopyridine, and 1,3,5-tris(1-phenyl-1H-benzimidazole-2-yl)benzene (TPBI, sublimed grade) were purchased from Tokyo Chemical Industries Co. Ltd. (Portland, OR, USA). Bathocuproine (2,9-dimethyl-4,7-diphenyl-1,10-phenanthroline, BCP) and *N*,*N*′-di(1-naphthyl)-*N*,*N*′-diphenyl-(1,1′-biphenyl)-4,4′-diamine (NPB, sublimed grade) were purchased from Sigma-Aldrich (St. Louis, MO, USA). Tetrabutylammonium hexafluorophosphate (TBAPF_6_) and ferrocene (Fc) were purchased from Sigma-Aldrich (St. Louis, MO, USA), and potassium carbonate (K_2_CO_3_) was purchased from Fisher Scientific (Fair Lawn, NJ, USA). Analytical grade chloroform (CHCl_3_), tetrahydrofuran (THF), hexane, ethyl acetate (EA), 1,4-dioxane, acetone, isopropanol, acetonitrile (ACN), and dichloromethane (DCM) were purchased from Macron (Center Valley, PA, USA). High-purity aluminum (Al) and calcium (Ca) with purity of 99.999% were purchased from Angstrom Engineering Inc. (Kitchener, ON, Canada). Glass slides coated with indium tin oxide (ITO) with sheet resistance of 8–12 Ohm square^−1^ were purchased from Delta Technologies (Loveland, CO, USA). Flash column chromatography was performed on silica gel (Sorbent Technologies, 60 Å, 40–63 μm) slurry packed into glass columns. Deionized water was obtained from an Elga model PURELAB ultra water-filtration system. 

### 2.2. Instrumentation

A scanning UV-vis spectrophotometer (UV-3101PC, Shimadzu, Columbia, MD) was used to obtain absorption spectra and a HORIBA Spex Fluorolog-3-spectrofluorometer (FL3-22TAU3, Jobin-Yvon, Edison, NJ) was used for steady-state fluorescent spectra, along with quartz cuvettes (Starna Cells, Atascadero, CA, USA) with path lengths of 1 cm (for solutions) and quartz slides for thin films (1mm, Ted Pella, Inc., Redding, CA, USA). The entrance and exit slit bandwidths of this spectrofluorometer were maintained at 3–5 nm for recording photoluminescence spectra. The same spectrofluorometer was used for photostability experiments with entrance slit bandwidth maintained at 14 nm. Absolute quantum yields of compounds were obtained using a Petite Integrating Sphere (Jobin-Yvon, Edison, NJ, USA) installed in this fluorometer. A PTI Time Master TM-11/2005 lifetime spectrometer (Photon Technology International, Edison, NJ, USA) was used in single photon counting experiments; LED excitation sources with wavelengths 297 nm (for compounds **B** and **C**) and 340 nm (for compound **A**) were used in these experiments. Thin films (75 ± 7 nm thickness) of compounds **A**, **B**, and **C** were prepared for spectroscopic characterizations on clean quartz slides (Ted Pella, Inc., Redding, CA, USA) by spin coating, using model WS-650MZ-23NPPB spin-coater (Laurell Technologies Corporation, North Wales, PA, USA). For spin coating, dilute chloroform solutions (0.1–0.5 mM) were filtered through syringe filters (0.1 µm pore size), and were spin-coated on clean quartz slides (100 µL solution volume at 1500–2000 rpm, with 2 min spinning duration). A Hi Res Modulated TGA 2950 Thermogravimetric Analyzer (TA Instruments, New Castle, DE, USA) was employed to obtain thermal decomposition data. An Autolab potentiostat (model PGSTAT 302N, Metrohm, Riverview, FL, USA) was used for cyclic voltammetry analysis at room temperature, using a three-electrode system consisting of a platinum disk (3 mm diameter) working electrode, Ag/AgNO_3_ non-aqueous reference electrode, and a Pt wire counter electrode (CH Instruments, Austin, TX, USA). The reference electrode was checked against ferrocene (Fc) standard each time before and after experiments were performed, and the measured potentials were reported against the Fc/Fc^+^ redox potential value. For cyclic voltammetry experiments, ACN or DCM solutions of TBAPF_6_ (1 mM) were used as the supporting electrolyte. A Bruker Kappa APEX-II DUO diffractometer (Bruker, Madison, WI, USA) was employed to perform single crystal X-ray diffraction (XRD). A PANalytical Empyrean multipurpose diffractometer (Westborough, MA) with a copper anode was employed to obtain powder X-ray diffraction (PXRD) data. ^1^H NMR and ^13^C NMR spectra were recorded at 400 MHz and 100 MHz, respectively, and are reported in ppm downfield from tetramethylsilane. High-resolution mass spectra (HRMS) were obtained at the Louisiana State University Mass Spectrometry Facility using an Agilent 6230 ESI TOF and Bruker UltrafleXtreme MALDI TOF instruments.

OLED prototypes were fabricated using vacuum thermal deposition. An ultra-high vacuum thermal evaporator (Nexdep series, mounted in a glovebox, Angstrom Engineering, Kitchener, ON, Canada) was used to deposit metal and organic layers on ITO-coated glass substrates using a previously reported procedure [28]. In brief, ITO-coated glass substrates were cleaned by sequential ultra-sonication in an aqueous detergent solution, DI water, acetone, and isopropanol. Then, these cleaned substrates were dried overnight inside a glovebox and subjected to oxygen plasma treatment for 20 min under ambient conditions. These oxygen plasma treated ITO coated glass substrates were returned to the glovebox to prepare OLED prototypes by mounting them onto the sample holder inside the VTE chamber. The base pressure of the VTE system was maintained at < 1 × 10^−6^ Torr throughout the material deposition process. Depositions of metals and organic layers were performed through specially designed shadow masks with rates of 1 Å s^−1^ (organic compounds), 0.3 Å s^−1^ (Ca), and 2 Å s^−1^ (Al). A Bruker Dektak XT stylus profilometer (Bruker Nano Inc., Tucson, AZ, USA) was used to determine and calibrate the OLED layer thicknesses. Electroluminescence spectra and performance characteristics of the OLED prototypes were obtained using a PTI QuantaMaster4/2006SE spectrofluorometer (Photon Technology International, Edison, NJ) combined with an integrating sphere (Labsphere, North Sutton, NH, USA). A prototype OLED device was attached to an optical port of the integrating sphere using a specially fabricated Teflon holder. A Keithley 2601 source meter (Tektronix, Inc., Beaverton, OR, USA) was employed to control and measure the current and voltage of OLED prototypes. The absolute total spectral flux measurement was calibrated using a SCL-050 lamp standard (Labsphere, North Sutton, NJ, USA). 

### 2.3. Computational Studies

All DFT and time-dependent DFT computations (at B3LYP/6-31G(d) level of theory) were carried out using Gaussian 16 computational package running on a Windows-based computer [29]. The geometry optimization was done in gas phase, and frequency calculations were performed on each optimized structure to ensure it was a true minimum. 

## 3. Synthesis and Characterization

Synthesis of compound **A** is described here to provide a representative protocol. An Airfree flask was charged with 2-bromo-7-*tert*-butylpyrene **P1** (341 mg, 1.00 mmol), 1-phenyl-2-[3-(4,4,5,5-tetramethyl-1,3,2-dioxaborolan-2-yl)phenyl]-1*H*-benzimidazole **P2** (397 mg, 1.01 mmol), and tetrakis(triphenylphosphine)palladium(0) (120 mg, 0.1 mmol) in a nitrogen atmosphere. Next, 1,4-dioxane (degassed, 80 mL) and an aqueous potassium carbonate solution (degassed, 0.2 M, 20 mL) were added to the same flask. The reaction mixture was stirred at 60 °C for 24 h under argon atmosphere in a sealed flask. Crude product precipitated inside the flask as white needles upon cooling the reaction mixture to room temperature. The crude product was isolated using vacuum filtration, followed by air-drying at ambient temperature. Silica gel flash column chromatography purification was performed on the crude product using ethyl acetate: hexane (2:3 v/v) as an eluent to isolate pure compound **A** as colorless needles (332 ±37 mg, yield 58%). 1H NMR (CD_2_Cl_2_, 400 MHz, ppm): δ 8.31 (s, 2H), 8.14 (m, 4H), 8.09 (m, 2H), 8.04 (m, 1H), 7.93 (m, 2H), 7.83 (d, 1H), 7.71 (m, 3H), 7.59 (t, 1H), 7.51 (m, 2H), 7.40 (m), 7.35 (m, 2H), 1.64 (s, 9H); 13C Proton Decoupled NMR (CD_2_Cl_2_, 100 MHz, ppm): δ 152.1, 149.4, 143.3, 141.3, 137.6, 137.5, 131.4, 131.0, 130.8, 130.1, 129.1, 128.7, 128.7, 128.4, 128.0, 127.8, 127.2, 123.8, 123.3, 123.2, 122.8, 122.5, 119.7, 110.5, 35.2, 31.6. HRMS (ESI-TOF) m/z 527.2417 [M+H]+ (calcd. for C_39_H_30_N_2_ 527.2409). 

A single crystal of the compound **A** obtained by crystallization of the reaction mixture was analyzed using X-ray crystallography. The resolved structure included solvent 1,4-dioxane co-crystallized with the compound **A**. Crystal Data for Compound **A** (C_39_H_30_N_2_ + C_4_H_8_O_2_) are; (M = 614.75 g/mol): monoclinic, space group P2_1_/n (no. 14), a = 19.5670(4) Å, b = 6.09880(10) Å, c = 27.1657(6) Å, β = 98.2182(13)°, V = 15,588.1(3) Å^3^, Z = 4, T = 100.0(5) K, μ(CuKα) = 0.604 mm^−1^, Dcalc = 1.273 g/cm^3^, 31690 reflections measured (5.2° < 2θ < 136.6°), 5870 unique (Rint = 0.0404) that were used in all calculations. The final R1 was 0.0391 (I > 2σ(I)) and wR2 was 0.1048 (all data). CCDC 1902159 contains the supplementary crystallographic data for this paper in CIF format. These data can be obtained free of charge via http://www.ccdc.cam.ac.uk/conts/retrieving.html (accessed on 23 September 2021), or from the CCDC, 12 Union Road, Cambridge CB2 1EZ, UK; Fax: +44 1223 336033; E-mail: deposit@ccdc.cam.ac.uk.

For the synthesis of compound **B**, the molar ratio of 1,3-dibromo-7-tert-butylpyrene **P3** and 1-phenyl-2-[3-(4,4,5,5-tetramethyl-1,3,2-dioxaborolan-2-yl)phenyl]-1H-benzimidazole **P2** used was 1.00:2.05. For the synthesis of compound **C**, the molar ratio of 1,3,6,8-tetrabromopyrene **P4** and 1-phenyl-2-[3-(4,4,5,5-tetramethyl-1,3,2-dioxaborolan-2-yl)phenyl]-1H-benzimidazole **P2** used was 1.00:4.05. The detailed synthesis protocols for compound **B** and compound **C** are provided in the Supporting Information. Compound **B** was a yellow solid (yield 67%) and compound **C** was a light brown solid (yield 65%). Complete characterization data for **B** and **C** are provided in the Supporting Information.

## 4. Results and Discussion

### 4.1. Solid-State Morphology 

**Figure 2:**
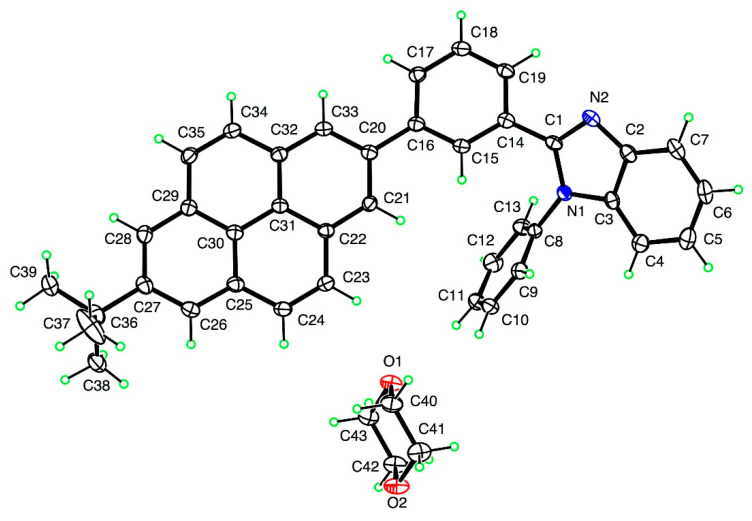
Single-crystal XRD derived ORTEP diagram for compound **A**. The structure includes co-crystallized solvent 1,4-dioxane.

Compounds **A**, **B**, and **C** were synthesized using Suzuki coupling protocol between respective mono-, di-, or tetrabromopyrenes (**P1**, **P3**, or **P4**) and 1-phenyl-2-[3-(4,4,5,5-tetramethyl-1,3,2-dioxaborolan-2-yl)phenyl]-1H-benzimidazole **P2** (Figure 1). Powder and/or single-crystal XRD experiments were performed for compounds **A**, **B**, and **C** (Figure 2 and Figure S1 in the Supporting Information). The powder XRD data indicates that compounds **A**, **B**, and **C** are predominantly amorphous, as their XRD plots display two intense broad scattering bands at 2θ approx. 10° and 20°, while displaying systematic reduction of the intensity of sharp Bragg diffraction peaks stemming from the crystalline phase (the Bragg peaks essentially disappear for the compounds **B** and **C**). Crystallinity, determined as a ratio of integrated intensity of the Bragg diffraction peaks to the total integrated intensity (Bragg peaks plus diffuse background) [30], was estimated at 40% for a compound **A** sample, but only 5% for compound **B**, and zero—for compound **C**. The observed decreasing crystallinity trend (i.e., **A** >> **B** > **C**) is correlated with a systematic increase in the number of phenyl and/or 1-phenylbenzimidazole substituents in these compounds (i.e., 3, 5, and 8 substituents in compounds **A**, **B**, and **C**, respectively). An increasing number of large substituents hinders crystalline packing and disrupts π stacking of the pyrenyl moieties by elevating steric hindrance, thus making the compounds more amorphous in solid state. Generally, amorphous organic compounds are more suitable for optoelectronic applications than crystalline compounds because amorphous compounds lack non-linear optical, thermal, and electronic characteristics stemming from crystalline anisotropy [31,32].

Since only compound **A** showed reasonable crystallinity in powder XRD data, we were able to obtain acceptable quality single crystals, and single-crystal X-ray structure was determined for the compound **A** (Figure 2). 

Interestingly, solid-state packing of compound **A** (Figure S2 in the Supporting Information) indicated that two neighboring **A** molecules are paired in a head-to-tail fashion, with the closest intermolecular distance between two neighboring benzimidazole N atoms being 5.5 (±3) Å. It is noted that among aliphatic and aromatic moieties attached to a pyrene core to induce steric hindrance, the phenyl group that is attached to the N atom (position 1) of benzimidazole unit is the most twisted moiety in the molecule. This phenyl group is positioned nearly orthogonal to the rest of the molecule as shown in Figure 2, thus causing additional twisting of the benzimidazole unit and resulting in the highest steric hindrance to prevent stacking aggregation in the solid state. In addition to causing significant steric restrictions for crystalline packing in the solid state, the bulky non-symmetrical 1-phenylbenzimidazole substituents may also induce existence of different conformational isomers in solid state, that can also contribute to increasing amorphous character of the solid-state phase. Since the number of benzimidazole-attached phenyl groups increases from **A** to **B**, and to **C**, solid-state packing is drastically affected, as denoted by a shift in the solid-state morphologies from more crystalline (compound **A**) to completely amorphous (compound **C**). 

### 4.2. Photo and Thermal Stability 

Organic blue emitters may undergo various degradation processes that affect them to different extents [33,34,35,36,37,38,39,40]. OLED degradation is caused by external factors such as heat, light, moisture, and oxygen. Internal factors that cause OLED degradation include fabrication errors such as formation of pinholes and/or deformities, morphological changes that may occur in organic layers, and excessive electrical stress in non-optimized designs. Some organic materials are less chemically stable. For example, it has been reported that blue phosphorescent emitters with strong electron withdrawing moieties (i.e., F, CN) and with iridium metal centers are more susceptible to degradation when used in optoelectronic devices [33,34,35,36]. Some polymeric blue emitters are also susceptible to delamination and/or non-emissive ‘black’ spot formation as a result of morphological changes [37]. Heat generation as a result of OLEDs biasing due to Joule heating stems from a high resistance of organic layers, and non-radiative recombination also contributes to OLED degradation [38]. Photodegradation of OLED emitters is induced by light in the presence of oxygen, and may occur during material handling, device fabrication, and device operation [39,40]. Therefore, among the aforementioned factors that may potentially lead to OLED degradation, susceptibility to photo- and thermal degradation was evaluated for compounds **A**, **B**, and **C** as a preliminary assessment of the stability of these compounds and their suitability for OLED applications.

**Figure 3:**
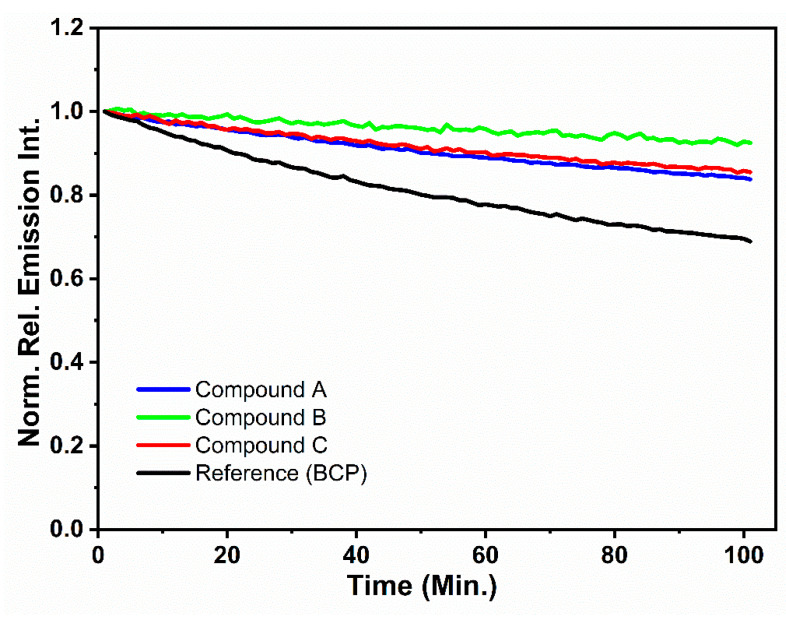
Time-dependent relative photoluminescence intensity change curves for compounds **A**, **B**, and **C** as thin films irradiated over a time period of 100 min.

Photostability of compounds **A**, **B**, and **C** was evaluated using a previously reported time-dependent kinetic fluorescence method [41]. In brief, thin films of compounds **A**, **B**, and **C** on quartz plates were intensively irradiated with monochromatic light at their respective maximum absorbance (*λmax*) wavelengths for 100 consecutive minutes, while recording the photoluminescence intensity fluctuations at the respective wavelengths corresponding to emission maxima. Accordingly, *λmax* values used in this study for compounds **A**, **B**, and **C** were 347, 372, and 400 nm, respectfully. It is assumed that any decrease in the recorded emission intensity with increasing irradiation time is correlated to the extent of photodegradation of the thin-film material. Accordingly, the percentage of photodegradation for all compounds was estimated using Equation (1).

(1) $$Photodegradation\;\left(\%\right)=\left(1-\frac{I}{I_{0}}\right)\times 100\;\%$$

where *I* is the emission intensity after intense irradiation of the thin films for a sufficient time period to undergo substantial photodegradation and *I*_0_ is the initial emission intensity (prior to irradiation). Bathocuproine (BCP), which is a well-known electron transport/hole blocking compound, was used as the reference compound [39]. Resultant photodegradation curves are presented in Figure 3. Under these experimental conditions, the reference BCP showed the highest photodegradation, with an approximately 30% reduction of the initial fluorescence intensity under the given experimental conditions (Figure 3). This high photobleaching rate observed for BCP can be attributed to the high-energy excited state of BCP, as indicated by its large HOMO–LUMO energy gap value, which leads to a high susceptibility towards photo-induced degradation reactions [39]. Compounds **A** and **C** showed a 16% and a 14% reduction of relative fluorescence intensity as compared to BCP. In contrast, compound **B** displayed the lowest photobleaching, with only 7% of relative fluorescence intensity decay among tested compounds under given experimental conditions. Therefore, it can be assumed that compounds **A**, **B**, and **C** were reasonably photostable with respect to the reference compound BCP. The exact structural reasons for the observed relative photostability trend of compounds **A**, **B**, and **C** (**A** ≤ **C** < **B**) are not clear. We notice that the LUMO energy in these compounds decreases in the order of increasing photostability (–2.23 eV for A, –2.31 eV for C, and –2.56 eV for B, Table 4). The LUMO energy may affect excited state reactivity of these compounds towards oxygen [42], resulting in faster photooxidation rates for A and C. Photostability of organic compounds generally depends on the interplay of many complex factors and susceptibility towards various photobleaching mechanisms that are not yet fully understood [43]. 

Resistance to thermal decomposition is vital for OLED emitters [35,38]. Therefore, thermal stability of compounds **A**, **B**, and **C** was evaluated using thermogravimetric analysis (TGA) [44]. Typical TGA experiments were conducted by heating a compound sample (<5 mg) in nitrogen atmosphere from 25 to 600 °C at a constant rate (10 °C min^−1^). Since thermal degradation of most organic compounds is associated with the formation of volatile compounds, the temperature that corresponds to an onset of weight loss (<5%) is reported as the onset decomposition temperature of the compound (*T*_onset_), and it is determined by using a step-tangent method [44]. The resultant TGA profiles of compounds **A**, **B**, and **C** are provided in Figure S5 in the Supporting Information, and *T*_onset_ values are listed in Table 1. Accordingly, compounds **A**, **B**, and **C** displayed substantial thermal stability with *T*_onset_ values in the range of 308–467 °C. The *T*_onset_ trend for the compounds, **C** < **B** < **A**, can be attributed to a gradual decrease of the relative fraction of the highly thermally stable pyrenyl moiety in compounds **A**, **B**, and **C** (i.e., 38, 25, and 16%, respectively). Accordingly, compound **A** with the highest percentage of pyrenyl fraction (38%) also showed the highest *T*_onset._ In contrast, compound **C** with the lowest percentage of pyrenyl fraction (16%) showed the lowest *T*_onset_ among these three compounds. 

### 4.3. Spectral Properties in Solution and in Solid State

**Figure 4:**
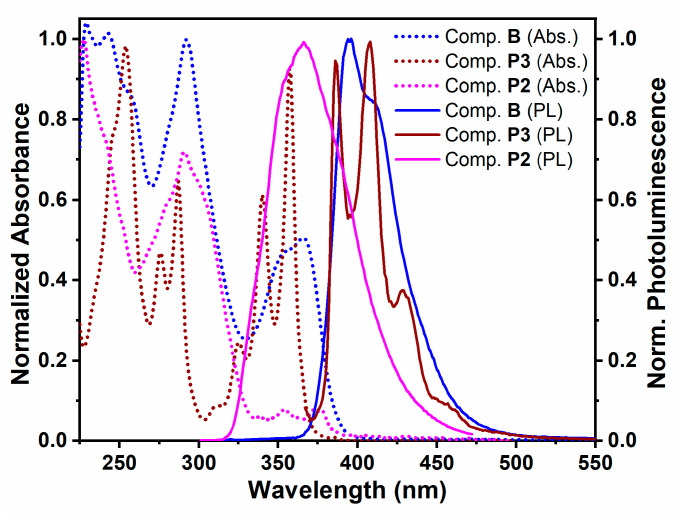
Normalized UV-vis absorption (Abs.) and photoluminescence (PL) spectra of compound **B** and its synthetic precursors: pyrene derivative (**P3**) and benzimidazole derivative (**P2**) in DCM.

Normalized UV-vis absorption and photoluminescence spectra of compound **B** and synthetic precursors of compound **B**, i.e., pyrene derivative (**P3**) and benzimidazole derivative (**P2**), in DCM solution (5–10 µM) are presented in Figure 4. Similarly, absorption and photoluminescence spectra of compounds **A** and **C**, and their respective synthetic precursors were recorded in DCM (5–10 µM) and are shown in Figures S3 and S4 in the Supporting Information. It is noted that spectral characteristics of compounds **A**, **B**, and **C** are complex and could be attributed to both pyrene and benzimidazole components (Figure 4 and Figures S3 and S4 in the Supporting Information). For example, absorption and photoluminescence spectral features of compound **B** show similarities to those of its synthetic precursors (Figure 4). The absorption spectrum appears as a superposition of the pyrene and benzimidazole spectral features. Nevertheless, detailed features in the spectrum are unique in terms of shape, relative peak intensities, and peak positioning. For example, the absorption spectrum of compound **B** shows a much less pronounced fine vibronic structure compared to the spectra of its synthetic precursors. The additive characteristic of the absorption spectrum of compound **B** clearly indicates low extent of π-electron delocalization between pyrene and benzimidazole chromophores so that the two chromophores behave as electronically isolated entities in the molecule of **B**. This observation is also confirmed using the X-ray single crystal structure of the related compound **A** where a significant twist can be found between the pyrene and benzimidazole units in the molecular structure (Figure 2 and Figure S2 in the Supporting Information). In contrast to the additive characteristic of the absorption spectrum, the photoluminescence spectrum of compound **B** closely resembles the pyrene emission band (albeit with a less pronounced vibronic structure), and there was no benzimidazole emission band observed (Figure 4). This could be explained by considering an efficient intramolecular excitation energy transfer between the higher-energy benzimidazole and the lower-energy pyrene chromophores via the dipole-induced dipole Förster mechanism. Indeed, there is a strong overlap between the emission band of the benzimidazole compound **P2** and the absorption band of the pyrene compound **P3**, that should facilitate energy transfer by this mechanism. 

**Figure 5:**
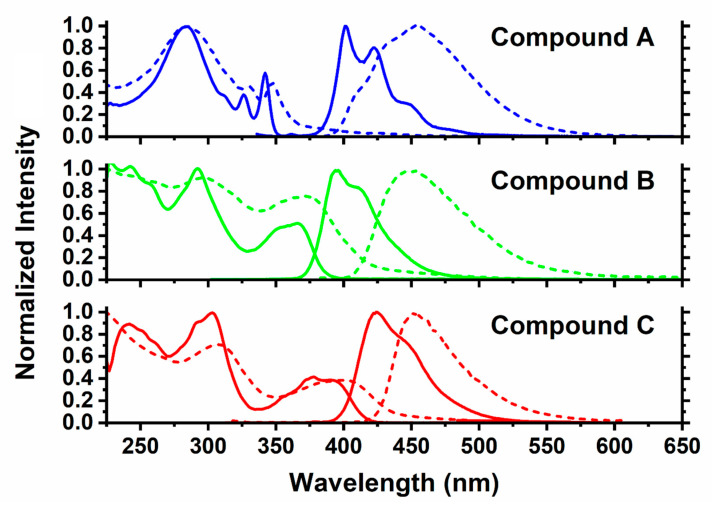
Normalized UV-vis absorption and fluorescence spectra of compound **A**, **B**, and **C** in DCM solutions (1 µM, solid lines) and neat films (dashed lines).

Figure 5 shows a comparison of the normalized absorption and photoluminescence spectra of pyrene-benzimidazole derivatives (compounds **A**, **B**, and **C**) in dilute DCM solutions (1 µM–10 µM), as well as in thin films on quartz. All three compounds showed multiple absorption peaks corresponding to multiple electronic transitions, with or without distinguishable vibronic features (i.e., shoulders, peak clusters). The absorption maxima (*λ*_max_) values for compounds **A**, **B**, and **C** are summarized in Table 1 for solution and solid states. It is noteworthy that a systematic red-shift of *λ*_max_ values was observed for compounds **A**, **B**, and **C**, in particular for the lowest energy absorption band. This could be due to the increasing contribution of the extended conjugation in these compounds with the increasing number of benzimidazole units. In solid-state, the absorption spectra were broadened as denoted by the increase in the full lengths of half maxima (FWHM) values. For example, FWHM values of the lowest energy absorption bands were broadened by 12–21 nm for compounds **A**, **B**, and **C** in the solid-state relative to solution (Table 1). In addition, the solid-state absorption bands were red-shifted. For example, the lowest energy absorption bands were bathochromically shifted by 4–6 nm for compounds **A**, **B**, and **C** in the solid-state as compared to the corresponding solution spectra (Table 1). These spectral changes in the solid-state suggest that despite the steric hindrance caused by the bulky substituent groups, compounds **A**, **B**, and **C** still demonstrated some propensity (albeit not very strong) towards aggregation in the solid state.

Combined photoluminescence spectra for compounds **A**, **B**, and **C** are also presented in Figure 5 for both solution and solid states, and a summary of photoluminescence characteristics is provided in Table 2. As discussed above, photoluminescence spectra of all three compounds display a single broad band corresponding to emission from the pyrene chromophore. While this band shows a distinct vibronic structure in the solution state, no such structure was observed in the solid-state, suggesting noticeable pyrene chromophore aggregation in the solid-state [45]. Emission maxima (*λ*_max_) values recorded in DCM solution were found in the range of 395–424 nm. In solid-state, the *λ*_max_ values ranged from 452 nm to 456 nm (Table 2). Thus, the solid-state photoluminescence spectra of compounds **A**, **B**, and **C** were red-shifted relative to solution by 61 nm, 58 nm, and 30 nm, respectively. The values of *λ*_max_ in solid state for all three compounds were relatively close, resulting in systematic reduction of the Stokes shifts, and suggesting a smaller extent of aggregation for the compound **C** as compared to the compounds **A** and **B**. Simultaneously, a systematic reduction of peak broadening was observed in the solid-state, as indicated by a gradual decrease in FWHM values (81 nm, 72 nm, and 52 nm for the compounds **A**, **B**, and **C**, respectively). Hence, this further confirms a gradual reduction of the pyrene chromophore aggregation in the solid state, in agreement with the initial design plan discussed in Section 4.1. It is important to note that despite the observed bathochromic shift and broadening of the emission bands in the solid state, all three compounds displayed emission values that were characteristic of pyrene monomer and not of pyrene excimer. Thus, we can conclude that our design approach was viable for targeting minimization of intermolecular stacking, disruption of crystalline packing, and decreasing pyrene chromophore aggregation in compounds **A**, **B**, and **C**. 

As photoluminescence of these compounds originated from the non-aggregated pyrene chromophore emission, it was primarily confined to the violet-blue region (in DCM) and blue region (in solid-state) of the electromagnetic spectrum (EMS). The color of these compounds has been assigned in accordance to CIE coordinate values that are listed in Table 2. In summary, compounds **A**, **B**, and **C** have average CIE coordinates of (0.160 ± 0.005, 0.029 ± 0.009) in DCM solution and (0.152 ± 0.007, 0.126 ± 0.005) in solid-state. These CIE coordinates comply with the general criterion for blue emitters, where *y* < 0.150 and (*x* + *y*) < 0.300 [5]. These values slightly deviate from the National Television System Committee (NTSC) and European Broadcast Union (EBU) standards since these require average CIE coordinates of (0.150 ± 0.010, 0.07 ± 0.010) for blue emitters in electronic displays [4]. Nonetheless, many blue emitters of significant commercial interest (i.e., emitters that show high OLED device performance) often have CIE coordinates that are not fully compliant with NTSC/EBU standards [46,47,48]. Solid-state emission spectra of compounds **A**, **B**, and **C** also show negligible emission in the near-UV region of the EMS. Since electronic screens frequently interact with human eyes, the emission in the near-UV range from electronic screens can harm eyes through development of conditions such as corneal sunburn, pterygium, and cataract that can ultimately lead to blindness. Therefore, blue emitters that do not emit UV light, e.g., compounds **A**, **B**, and **C**, would be safer for human eyes if used in electronic displays [49].

The origin of the emission spectra as fluorescence stemming from the lowest-energy pyrene S_1_ state, and thus unlikely participation of a triplet T_1_ state was confirmed by DFT computational studies, and was corroborated in emission lifetime experiments *(vide infra)*. Specifically, geometry optimizations of compounds **A**, **B**, and **C** were carried out for the ground state singlet S_0_ and triplet T_1_ states using DFT computations at B3LYP/6-31G(d) level of theory, and the optimization of the excited state S_1_ was done using time-dependent DFT computations at the same level of theory. The results of the computational studies are summarized in Table 3. They indicate that there is a significant S_1_–T_1_ energy gap ranging from 1.26 eV for compound **A** to 0.93 eV for compound **C**. Such a large singlet-triplet energy gap would preclude both population of the T_1_ state through a forbidden intersystem crossing process, and participation of the T_1_ state in fluorescent emission process (e.g., in delayed fluorescence, etc.).

### 4.4. Photoluminescence Quantum Yield (PLQY) and Lifetime Measurements

PLQY is the ratio of emitted photons to absorbed photons for a fluorophore and is influenced by factors such as optical characteristics, molecular rigidity, and inter/intra molecular interactions [50]. It is noted that a dye with a high PLQY may not necessarily exhibit high electroluminescence when used as an emitter in an OLED device and may even not exhibit similar emission behavior (i.e., emission peak shape, intensity, position, efficiency, etc.). This may be due to the differences in emissive and/or quenching mechanisms of photo- and electroluminescence, influence of other semiconductor layers within the device, device architecture, as well as physical and electrical properties of the emissive layer [51,52]. Nevertheless, compounds with low PLQY values typically do not show efficient electroluminescence. Therefore, it is important to estimate PLQY values of compounds **A**, **B**, and **C**, to further understand the optical characteristics of these compounds and evaluate their suitability for OLED applications. Accordingly, absolute PLQY values were measured using an integrating sphere for solutions and thin films deposited on quartz slides [53]. The resultant PLQY values of compounds **A**, **B**, and **C** are presented in Table 2. It is noteworthy that all three compounds show substantial PLQY values in both solution and solid state, and they increase in the following order: **A** < **B** < **C**. 

We also studied emission lifetimes in dilute DCM solutions using a single photon counting method (Table 3). All three compounds displayed relatively short lifetimes consistent with normal fluorescence from an S_1_ state, and indicating unlikeliness of participation of the more exotic mechanisms involving triplet state (e.g., delayed fluorescence). There was a clear trend of decreasing emission lifetime in the order **A** > **B** > **C**; this trend was commensurate with the increasing number of benzimidazole substituents at the pyrene chromophore. The larger number of substituents could contribute to vibronically coupled non-radiative deactivation of the excited state, and thus reduce the emission lifetime.

Using the experimental values of photoluminescence quantum yields and emission lifetimes, we also calculated fluorescence rate constants *k*_fl_ (Table 3). There is a clear trend of increasing *k*_fl_ in the order **A** < **B** < **C,** which explains the experimentally observed trend in increasing PLQY in the same order. The PLQY values were lower in solid-state relative to dilute solution, suggesting that some quenching occurs in the solid-state due to chromophore aggregation, as was also revealed in the spectroscopic studies described above. 

### 4.5. Electrochemical Properties 

Equations (2) and (3) were used to calculate HOMO and LUMO energies of compounds **A**, **B**, and **C** from cyclic voltammetry (CV) data, which is a common experimental method for estimating the highest occupied molecular orbitals (HOMO) and the lowest unoccupied molecular orbitals (LUMO) energies of organic semiconductors [54]. The cyclic voltammograms obtained for compounds **A**, **B**, and **C** are presented in Figure S6 in the Supporting Information. The experimentally obtained energies of frontier molecular orbitals are listed in Table 3. All three compounds showed quasi-reversible oxidation peaks (Figure S6 in the Supporting Information). Furthermore, compounds **A** and **B** showed two distinguishable peaks with prominent shoulders. These multiple oxidation peaks can be attributed to separate oxidation of the imidazole and pyrene units. The electrochemical potential window for ACN (used for compounds **A** and **B**) was wider than DCM (used for compound **C**); therefore, only the first oxidation was recorded for the compound **C**. The onset of oxidation and reduction waves as obtained from cyclic voltammograms can be used for estimating oxidation (*E*_ox_) and reduction (*E*_red_) potentials [52]. For molecules with quasi-reversible cyclic voltammograms and showing only oxidation peak(s), the LUMO can be estimated using an optical energy gap (*E*_g_) and the HOMO values, which are obtained experimentally from CV data (Equations (2) and (3)).

(2) $$\mathrm{HOMO}\;\left(\mathrm{eV}\right)=-1e\;\left[E_{\mathrm{ox}}+4.71\right]\;V$$

(3) $$\mathrm{LUMO}\;\left(\mathrm{eV}\right)=E_{g\;}-\mathrm{HOMO}\;\left(\mathrm{eV}\right)$$

The *E*_g_ values for compounds **A**, **B**, and **C** were determined using UV-vis absorption spectra and hence referred to as an optical energy gap. This simple *E*_g_ calculation method is widely used for organic semiconductor materials through employment of Equation (4) [55]. This method is based on the hypothesis that the higher wavelength onset of the absorption spectrum corresponds to the minimum energy required to promote a ground state (HOMO) electron to the first excited state (LUMO), which is true for many small organic molecule semiconductors.

(4) $$E_{g}\;\left(\mathrm{eV}\right)=\mathrm{hf}=h\cdot \left(\frac{c}{\lambda_{onset}}\right)=\frac{1240}{\lambda_{onset}}$$

where *h* is Planck’s constant (6.626 × 10^−34^ J s), *c* is speed of light in vacuum (3.00 × 10^−8^ ms^−1^), and *λ_onset_* is the wavelength of the absorption onset (nm). The value of (*h* × *c*) is also a constant (1240 eV^.^ nm). Accordingly, HOMO and LUMO energies calculated using the aforementioned methods for compounds **A**, **B**, and **C** were estimated to be in the respective range of –5.10 to –5.56 eV and –2.23 to –2.56 eV (Table 4). It should be noted that *E_g_* gradually decreases with the increasing number of benzimidazole moieties, likely due to the more extended electronic conjugation. The experimental *E*_g_ values showed good correlation with the DFT calculated S_1_ excited state energies (Table 3). The HOMO and LUMO energies for these compounds were also in a range in which it would be relatively easy to find energetically matching and commercially available supporting organic semiconductors (i.e., charge transporters/injectors/blockers) and the work functions of most metal electrodes, making it easier to integrate these compounds into traditional OLED architectures [56].

### 4.6. Characterization of an OLED Prototype with Compound ***B*** as an Active Layer

Compound **B**, which exhibited the best overall combination of photo-physical characteristics, was used to fabricate an OLED prototype for preliminary evaluation of the OLED performance. Although using an electroluminescent material as dopant in a host matrix (doped device) may enhance the efficiency of OLED devices by controlling aggregation induced quenching, introducing favorable host-dopant energy transfer, and improving charge transport through the active layer, a non-doped device is simpler to fabricate, and it is also more reflective of electronic properties of the material. Thus, a non-doped OLED prototype was fabricated at this preliminary stage to reduce device complexity and study the electroluminescence characteristics of the emitter itself. 

**Figure 6:**
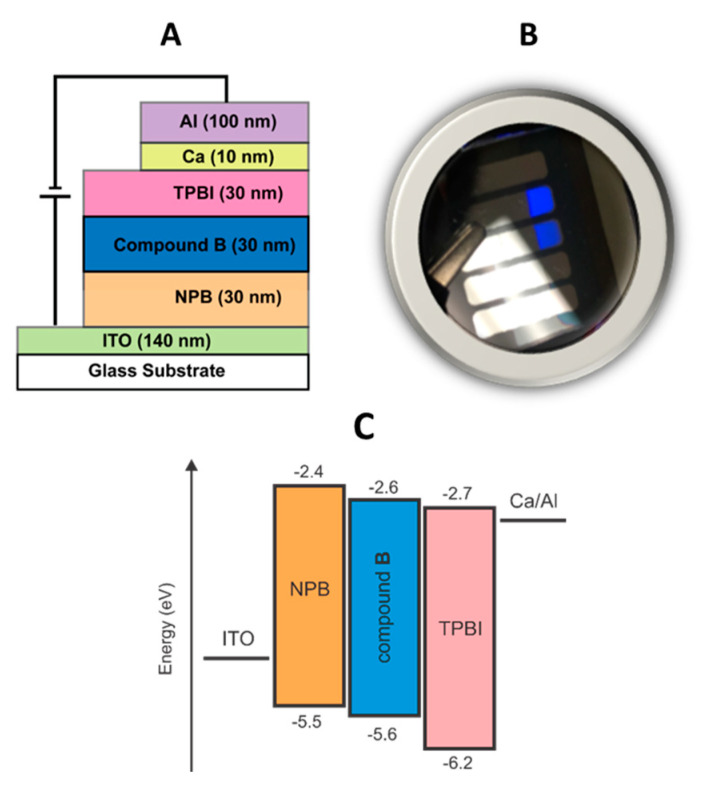
Structure of the OLED prototype (**A**), a photograph of an actual OLED showing blue emission at an applied voltage of 3 V (**B**), and the device energy diagram (**C**).

An optimized OLED prototype was fabricated using vacuum thermal deposition with the following device configuration: ITO (140 nm)/NPB (30 nm)/Compound **B** (30 nm)/TPBI (30 nm)/Ca (10 nm)/Al (100 nm), as schematically shown in Figure 6A. NPB is *N*,*N*′-di(1-naphthyl)-*N*,*N*′-diphenyl-(1,1′-biphenyl)-4,4′-diamine, and TPBI is 1,3,5-tris(1-phenyl-1H-benzimidazole-2-yl)benzene). Here, NPB and TPBI were used as hole and electron transport layers, respectively; ITO and Ca were electrode materials, and compound **B** was the non-doped emissive layer (the energy diagram of the device is shown in Figure 6C). In this device, no separate electron blocking layer was used, in order to examine electron and hole transporting ability of the compound **B**.

This OLED device showed a blue electroluminescence with *λ*_max_ at 454 nm, while turning on at 3 V. CIE coordinates of the electroluminescence spectrum were identical to that of the solid-state photoluminescence (0.1482, 0.1300), implying blue emission with substantial spectral purity. 

**Figure 7:**
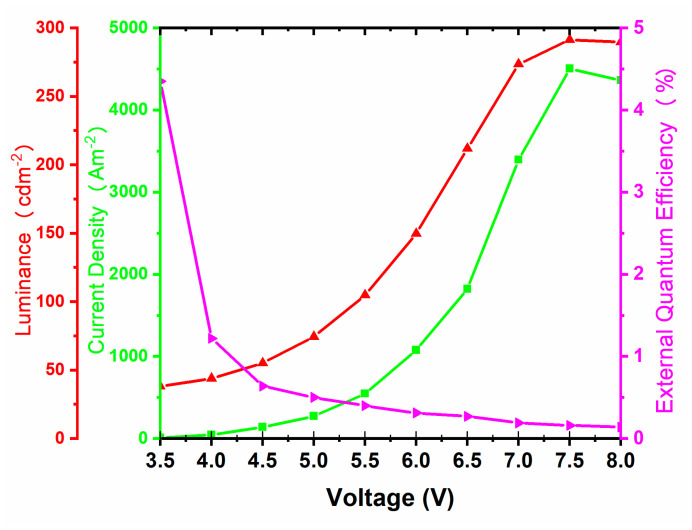
Performance plots for the OLED prototype with compound **B** as the emissive layer. Structure of the device is shown in Figure 6A.

The performance of this OLED prototype was assessed by determining current density, luminance, power and current efficiencies, and external quantum efficiency (EQE) in accordance with previously reported protocols [3,57]. At a typical for electronic devices applied voltage of 5.5 V, the EQE, which is the ratio of emitted photons into the viewing direction to injected charges, was recorded at 0.35 (± 0.04) %. At the same applied voltage, luminance, or the amount of light emitted per unit surface area of OLED weighed by the visual response of the human eye, was measured at 100 (± 6) cd m^−2^, and power and current efficiencies, which provide insights into energy consumption and light emitting ability of an OLED, were recorded as 1.2 (±0.6) lm W^−1^ and 0.17 (±0.2) cd A^−1^, respectively. The OLED performance plots of this prototype are provided in Figure 7. The observed luminance substantially increased upon increasing applied voltage, reaching 290 (±10) cd m^−2^ at 7.5 V. On the other hand, the EQE was at a maximum value of 4.3 (±0.3) % at 3.5 V and gradually decreased at the higher applied voltage primarily due to the rapid increase in the current density. These values are comparable to characteristics of the better blue OLED devices based on simple small-molecule organic chromophores. 

**Figure 8:**
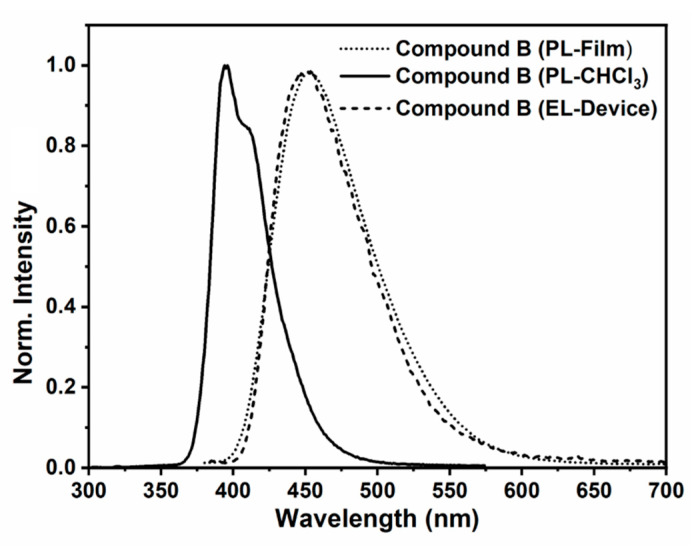
Normalized photoluminescence (PL) from solution and solid states, and electroluminescence from the OLED prototype recorded for compound **B**.

A comparison of photoluminescence (in DCM solution and in thin films) and electroluminescence (in the OLED prototype) of compound **B** is presented in Figure 8. It is noted that the luminescence in solid state (both photo- and electroluminescence) may be affected by the chromophore aggregation as denoted by the band broadening and red-shifting, as compared to the solution luminescence. Interestingly, this red-shifting of the luminescence band brought the emission precisely in the blue range, with a negligible overlap with near-UV region of the electromagnetic spectrum. It is noteworthy that the solid-state photoluminescence and electroluminescence were nearly completely superimposable, suggesting emission from the same pyrene chromophore regardless of the excitation method.

## 5. Conclusions

Three novel organic blue emitters were synthesized using Suzuki coupling. Compounds **A**, **B**, and **C** showed a systematic decrease in degree of crystallinity, as elucidated by powder X-ray diffraction analysis. Due to specific molecular design aimed at precluding extensive aggregation and preventing crystallization in solid state, these compounds displayed an essentially pure blue emission in the solid-state. This emission behavior was in stark contrast to many pyrene derivatives reported in the literature, which typically emit a bluish-green or greenish-blue fluorescence as a result of excessive solid-state aggregation and excimer formation. Spectroscopic characteristics, such as absorption, photoluminescence, and quantum yield, as well as electronic properties of these novel compounds were found suitable for optoelectronic applications. An OLED prototype, fabricated using compound **B** as the non-doped emissive layer, displayed a blue electroluminescence corresponding to CIE coordinates of (0.1482, 0.1300), with an external quantum efficiency of 0.35 (±0.4)% and luminance of 100 (±6) cd m^−2^ measured at 5.5 V. Future directions for this research will involve evaluation of OLED prototypes with doped emissive layers consisting of these materials by embedding them in a suitable host matrix to potentially improve the device performance, as well as fabrication and evaluation of the OLED prototypes with compounds **A** and **C**.

## Figures and Tables

**Table 1:** Thermal and absorption spectra data summary for compounds **A**, **B**, and **C**.

Compound	T_onset_ (°C)	*λ*_max_ (nm)	FWHM (nm)	ε × 10^4^ (M^−1^cm^−1^)	Compound	T_onset_ (°C)
		Sol ^x^	Film	Sol ^x^	Film	
**A**	467	284 326 342	285 329 347	43 12 8	73 18 20	5.7
**B**	378	243, 259 ^y^ 293 366, 351 ^y^	N/A 296 372	42 41 47	N/A 87 61	4.6
**C**	308	242 303, 293 ^y^ 378, 396 ^y^	N/A 309 400	58 44 53	N/A 55 74	3.7

^x^ In DCM solution (10 µM), ^y^ Prominent shoulder peak maxima, N/A: Not available within the scanned wavelength range.

**Table 2:** Summary of emission properties of compounds **A**, **B**, and **C**.

Compound	*λ*_max_ (nm)		FWHM (nm)		Stokes Shift (cm^−1^ × 10^3^)		PLQY		CIE Coordinates (*x,y*)	
	Sol ^x^	Film	Sol ^x^	Film	Sol ^x^	Film	Sol ^y^	Film	Sol ^x^	Film
**A**	395	456	45	81	3.9	6.9	48	35	0.1620, 0.0197	0.1483, 0.1214
**B**	396	452	43	72	2.1	4.8	71	51	0.1635, 0.0306	0.1482, 0.1300
**C**	424	454	49	52	1.2	3.0	98	56	0.1548, 0.0373	0.1600, 0.1275

^x^ In DCM solution (1µM), ^y^ Measured in acetonitrile: DCM (4:6 *v*/*v*) solvent mixture.

**Table 3:** DFT computational studies on singlet and triplet excited states^a^, lifetimes, and fluorescence rate constant *k*_fl_ for compounds **A**, **B**, and **C**.

Compound	Energy (eV) ^b^		S_1_–T_1_ Energy Gap (eV)	Lifetime *τ*_fl_ (ns) ^c^	*k*_fl_ (s) ^d^
	S_1_ State	T_1_ State			
**A**	3.37	2.11	1.26 ^e^	5.74	8.36 × 10^7^
**B**	3.12	1.94	1.18	4.12	1.72 × 10^8^
**C**	2.67	1.74	0.93	2.08	4.71 × 10^8^

^a^ The full geometry optimizations were carried out in gas phase using DFT (for S_0_ and T_1_ states) and time-dependent DFT (for S_1_ state) at B3LYP/6-31G(d) level of theory. ^b^ Relative to ground (S_0_) state. **^c^** Measured in DCM; excitation wavelength 340 nm (for compound **A**), and 297 nm (for compounds **B** and **C**). ^d^ Calculated as PLQY/*τ*_fl_. ^e^ The biexponential decay was detected, with a second component at 0.53 ns.

**Table 4:** Summary of the electrochemical properties of compounds **A**, **B**, and **C**.

Compound	*E*_g_ (eV)	HOMO (eV)	LUMO (eV)
Compound **A** ^y^	3.16	−5.39	−2.23
Compound **B** ^y^	3.00	−5.56	−2.56
Compound **C** ^z^	2.79	−5.10	−2.31
BCP	3.65 ^x^	−6.61 ^x^	−2.95 ^x^

^x^ These values were obtained from [56]. ^y^ In ACN solvent. ^z^ In DCM solvent.

## Data Availability

Not applicable.

## Supplementary Materials

The following are available online. Figure S1. Powder XRD data for comp; Figure S2. Molecular packing of compound A in the unit cells derived from single-crystal XRD; Figure S3. Normalized UV-vis absorption (Abs.) and photoluminescence (PL) spectra of compound A, and its parent compounds: pyrene derivative (P1) and benzimidazole derivative (P2) in DCM; Figure S4. Normalized UV-vis absorption (Abs.) and photoluminescence (PL) spectra of compound C, and its parent compounds: pyrene derivative (P4) and benzimidazole derivative (P2) in DCM; Figure S5. TGA profiles of compounds A, B, and C; Figure S6. Cyclic voltammograms of compounds A, B, and C in 0.1 M TBAPF6 in CH2Cl2/acetonitrile (potential vs. Fc/Fc+).

## Sample Availability

Samples of the compounds are not available from the authors.
