# Supplementary material for: Pyrene-Benzimidazole Derivatives as Novel Blue Emitters for OLEDs
Source: Molecules. 2021 Oct 28;26(21):6523. doi: 10.3390/molecules26216523 (PMC8588490; doi:10.3390/molecules26216523)
Supplement: Supplementary file 1 [file molecules-26-06523-s001.zip › molecules-1409078-supplementary.pdf]

Supplementary Information

Synthesis of compound B

An Airfree flask was charged with 1,3-dibromo-7-*tert*-butylpyrene **P3** (416 mg, 1.00 mmol), 1-phenyl-2-[3-(4,4,5,5-tetramethyl-1,3,2-dioxaborolan-2-yl)phenyl]-1*H*-benzimidazole **P2** (412 mg, 2.05 mmol), and tetrakis(triphenylphosphine)palladium(0) catalyst (120 mg, 0.1 mmol) in a nitrogen atmosphere. Next, 1,4-dioxane (degassed, 80 mL) and an aqueous potassium carbonate solution (degassed, 0.2 M, 20 mL) were added to the same flask. The reaction mixture was stirred at 60 °C for 24 h under argon atmosphere in a sealed flask. Crude product precipitated inside the flask as yellow solid upon the completion of the reaction. The crude product was isolated using vacuum filtration, followed by air-drying at an ambient temperature. The crude product was dissolved in chloroform and washed several times with brine solution and DI water, and then dried using anhydrous magnesium sulfate. The solvent was removed in vacuo, and the crude product was purified using flash column chromatography on silica gel (eluent hexanes: ethyl acetate, 2:3 v/v) three times. After solvent evaporation, compound **B** was obtained as a yellow powder (yield 67 %).

Synthesis of compound C

An Airfree flask was charged with 1,3,6,8-tetrabromopyrene **P4** (518 mg, 1.00 mmol), 1-phenyl-2-[3-(4,4,5,5-tetramethyl-1,3,2-dioxaborolan-2-yl) phenyl]-1*H*-benzimidazole **P2** (1605 mg, 4.05 mmol), and tetrakis(triphenylphosphine)palladium(0) catalyst (180 mg, 0.15 mmol) in a nitrogen atmosphere. Next, 1,4-dioxane (degassed, 80 mL) and an aqueous potassium carbonate solution (degassed, 0.2 M, 25 mL) were added to the same flask. The reaction mixture was stirred at 60 °C for 24 h under argon atmosphere in a sealed flask. Crude product precipitated inside the flask as a yellow solid upon the completion of the reaction. The crude product was isolated using vacuum filtration, followed by air-drying at an ambient temperature. The crude product was dissolved in chloroform and washed several times with brine solution and DI water, and then dried using anhydrous magnesium sulfate. The solvent was removed in vacuo, and the crude product was purified using flash column chromatography on silica gel (eluent hexanes: ethyl acetate, 2.5:3 v/v) three times. After solvent evaporation, compound **C** was obtained as a light brown powder (yield 65%).

Characterization of compounds B and C - ^{1}H, ^{13}C NMR, ESI-MS/MALDI-MS data

Compound B: ^{1}H NMR ($CDCl_3$, 400 MHz, ppm): δ 8.24 (s, 2H), 7.96 (q, 6H), 7.89 (m, 2H), 7.76 (m, 2H), 7.62 (m, 4H), 7.53 (m, 3H), 7.39 (m, 11H), 7.30 (d, 3H), 1.63 (s, 9H); ^{13}C Proton Decoupled NMR (CD_2Cl_2, 100 MHz, ppm): δ 152.3, 149.6, 143.2, 140.9, 137.5, 137.1, 136.1, 133.4, 132.4, 132, 131.9, 131.7, 131.6, 131.0, 130.5, 130.0, 129.1, 128.7, 128.6, 128.5, 128.4, 128.4, 128.0, 127.7, 127.6, 125.0, 124.5, 123.3, 123.1, 122.8, 122.5, 119.7, 110.5, 35.1, 31.6. HRMS (ESI-TOF) *m/z* 795.3592 $[M+H]^+$ (calcd. for $C_{58}H_{42}N_4$ 795.3409).

Compound C: ^{1}H NMR ($CDCl_3$, 400 MHz, ppm): δ 7.93 (d, 3H), 7.87 (m, 3H), 7.81 (s, 3H), 7.78 (s, 3H), 7.70 (m, 2H), 7.62 (m, 5H), 7.58 (m, 2H), 7.51 (m, 10H), 7.42 (m, 6H), 7.37 (m, 10H), 7.30 (m, 7H), 7.24 (m, 4H); ^{13}C Proton Decoupled NMR ($CDCl_3$, 100 MHz, ppm): δ 152.3, 143.1, 140.9, 137.3, 137.0, 136.3, 134.1, 132.2, 132.1, 132.0, 131.9, 131.8, 131.7, 130.5, 130.0, 130.4, 130.0, 129.6, 128.8, 128.6, 128.6, 128.4, 127.9, 127.5, 125.5, 125.2, 123.5, 123.1, 120.0, 110.6. MS (MALDI-TOF) *m/z* 1274.661 $[M^+]$ (calcd. for $C_{92}H_{58}N_8$ 1274.4784).

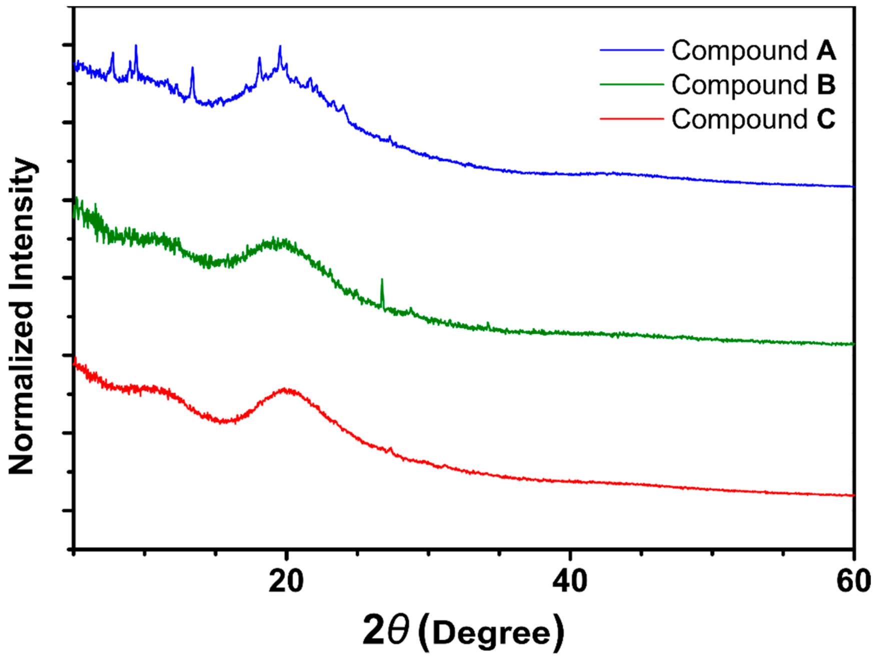

Figure S1. Powder XRD data for compounds **A**, **B**, and **C**.

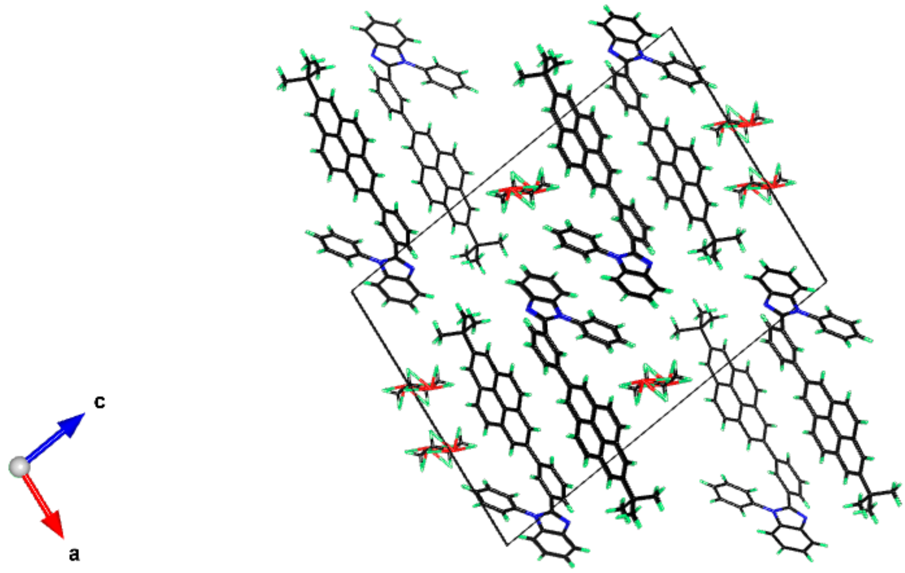

Figure S2. Molecular packing of compound **A** in the unit cells derived from single-crystal XRD.

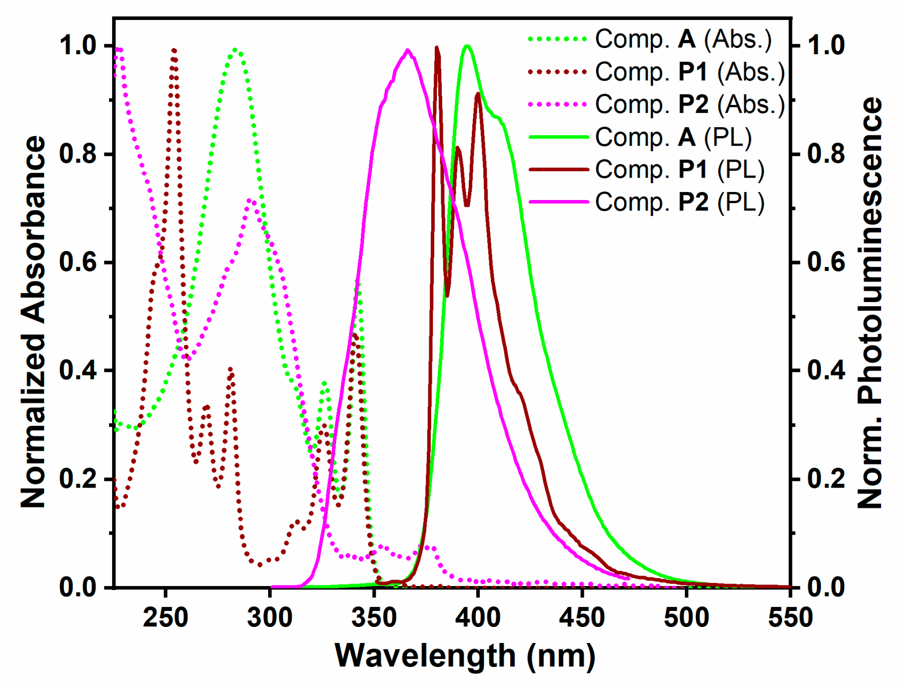

Figure S3. Normalized UV-vis absorption (Abs.) and photoluminescence (PL) spectra of compound **A**, and its parent compounds: pyrene derivative (**P1**) and benzimidazole derivative (**P2**) in DCM.

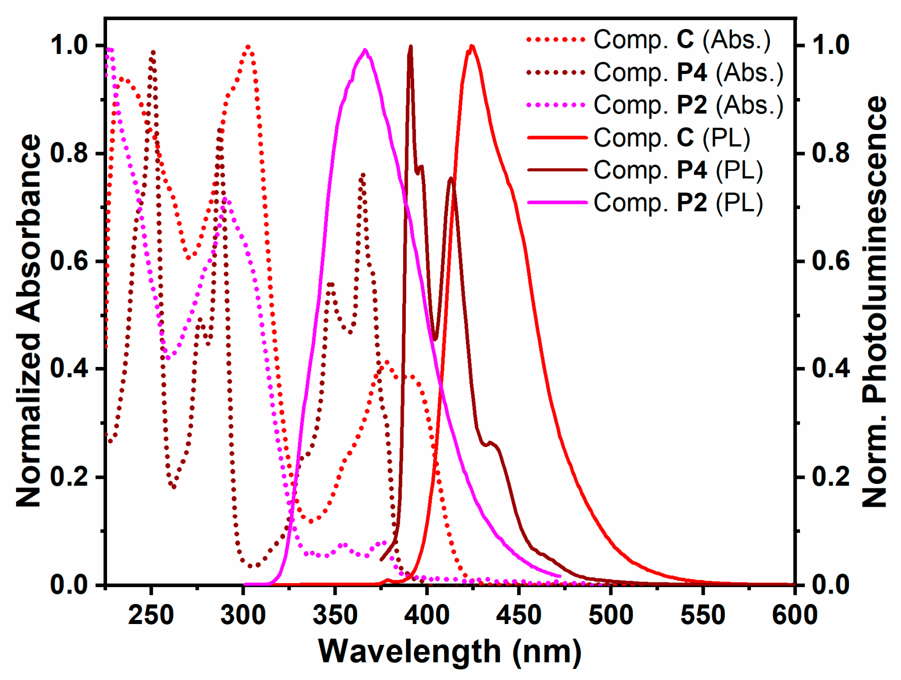

Figure S4. Normalized UV-vis absorption (Abs.) and photoluminescence (PL) spectra of compound **C**, and its parent compounds: pyrene derivative (**P4**) and benzimidazole derivative (**P2**) in DCM.

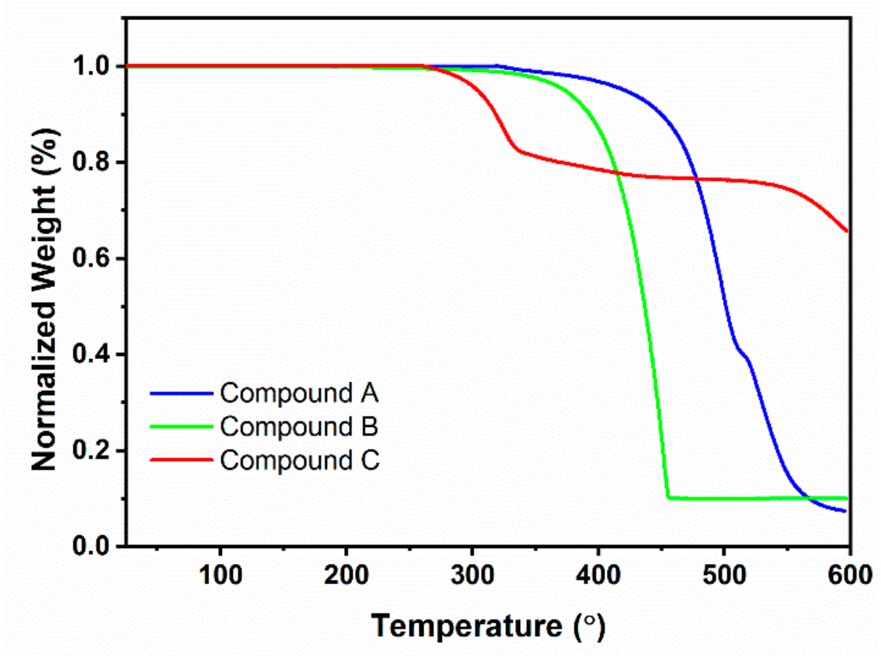

Figure S5. TGA profiles of compounds **A**, **B**, and **C**.

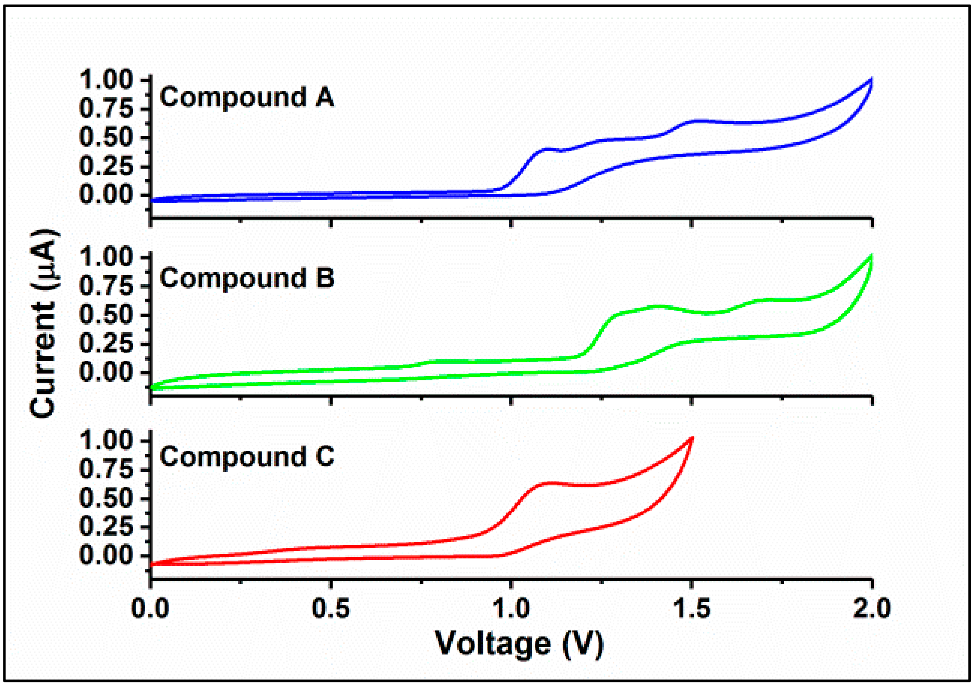

Figure S6. Cyclic voltammograms of compounds **A**, **B**, and **C** in 0.1 M $TBAPF_6$ in CH_2Cl_2/acetonitrile (potential vs. Fc/Fc^+).
